# Oxymatrine Inhibits Influenza A Virus Replication and Inflammation via TLR4, p38 MAPK and NF-κB Pathways

**DOI:** 10.3390/ijms19040965

**Published:** 2018-03-23

**Authors:** Jian-Ping Dai, Qian-Wen Wang, Yun Su, Li-Ming Gu, Hui-Xiong Deng, Xiao-Xuan Chen, Wei-Zhong Li, Kang-Sheng Li

**Affiliations:** 1 Department of Microbiology and Immunology, Shantou University Medical College, Shantou 515041, China; 16qwwang1@stu.edu.cn (Q.-W.W.); yunsu1978@163.com (Y.S.); 13lmgu@stu.edu.cn (L.-M.G.); 15hxdeng@stu.edu.cn (H.-X.D.); xxchen85@stu.edu.cn (X.-X.C.); liksedustdx@163.com (K.-S.L.); 2 Department of Veterinary Medicine, University of Maryland, College Park, MD 20742, USA; wzlistdxedu@sina.cn

**Keywords:** Oxymatrine, influenza A virus, TLR, MAPK, NF-κB

## Abstract

Oxymatrine (OMT) is a strong immunosuppressive agent that has been used in the clinic for many years. In the present study, by using plaque inhibition, luciferase reporter plasmids, qRT-PCR, western blotting, and ELISA assays, we have investigated the effect and mechanism of OMT on influenza A virus (IAV) replication and IAV-induced inflammation in vitro and in vivo. The results showed that OMT had excellent anti-IAV activity on eight IAV strains in vitro. OMT could significantly decrease the promoter activity of TLR3, TLR4, TLR7, MyD88, and TRAF6 genes, inhibit IAV-induced activations of Akt, ERK1/2, p38 MAPK, and NF-κB pathways, and suppress the expressions of inflammatory cytokines and MMP-2/-9. Activators of TLR4, p38 MAPK and NF-κB pathways could significantly antagonize the anti-IAV activity of OMT in vitro, including IAV replication and IAV-induced cytopathogenic effect (CPE). Furthermore, OMT could reduce the loss of body weight, significantly increase the survival rate of IAV-infected mice, decrease the lung index, pulmonary inflammation and lung viral titter, and improve pulmonary histopathological changes. In conclusion, OMT possesses anti-IAV and anti-inflammatory activities, the mechanism of action may be linked to its ability to inhibit IAV-induced activations of TLR4, p38 MAPK, and NF-κB pathways.

## 1. Introduction

Influenza A virus (IAV) often causes extensive respiratory infection, and severe IAV infection usually leads to acute lung injury (ALI) and acute respiratory distress syndrome (ARDS). ALI/ARDS is one of the main causes of death in intensive care units (ICU) with a high mortality rate (35% to 45%) [1,2]. Currently, no specific medicine is available for the management of ALI/ARDS.

During infection, IAV can hijack an array of intracellular signaling cascades for their own benefit, such as TLRs, RIG-I, PKC/PKR, PI3K/Akt, MAPK and NF-κB signaling pathways [3]. IAV infection can increase the expressions of TLR3, TLR4, TLR7, TLR8, TLR9, MyD88, IRAK4 and TRAF6, the phosphorylations of Akt, MAPK, and the nuclear translocation of NF-κB p65 [4,5,6,7,8]. In severe IAV-infected or died patients, TLR3, TLR4, TLR7, and MyD88 are lastingly and highly expressed [9,10]. It has been reported that lasting activation of TLR3 is detrimental to IAV-induced acute pneumonia, conversely, IAV-infected TLR3^−/−^ mice have an unexpected survival advantage with significantly lower viral titer, less inflammatory mediators, and fewer pathological changes in lung [11,12]. TLR4 is reported to markedly cluster at the site of IAV-cell interaction on cytomembrane and determines IAV entry and tissue tropism through MyD88 expression and p38 MAPK activation [13]. Inactivated H5N1 avian influenza virus can induce severe oxidative stress and rapidly leads to ALI through TLR4-TRIF-TRAF6-NF-κB signaling [14]. TLR4^−/−^ mice are reported to be highly refractory to IAV-induced ALI, and TLR4 antagonist eritoran can decrease viral titer and IAV-induced lethality [15]. Activation of TLR7 is also necessary for efficient replication of IAV [16]. Several inhibitors of TLR7/8-MyD88 signaling can effectively suppress IAV replication and IAV-induced up-regulation of proinflammatory cytokines and matrix metalloproteinase (MMPs) [7]. Moreover, activation of TLRs-MyD88 signals are also essential for MMP-9 release and neutrophil excessive migration, both of which are two principal causes for ALI/ARDS [17]. In addition, PI3K/Akt and the downstream cascades of TLRs, such as MAPK and NF-κB pathways, are also required for IAV infection and proliferation [13,18,19,20,21].

Based on the fact that IAV-induced over-expressions of TLRs, MyD88, TRIF, TRAF6, and abnormal nuclear translocation of NF-κB p65 are essential for IAV infection, we have constructed a series of luciferase reporter plasmids based on the promoters of these genes and the nuclear response element of NF-κB p65. Using these plasmids, we have performed several times of drug screening assays, and found that sophora root (*Sophora flavescens* Aiton), a traditional herbal medicine which has been used as an antipyretic, diuretic, and anthelmintic agent in China for thousands of years, can significantly inhibit the TLRs-MyD88-NF-κB pathway. Oxymatrine (C_15_H_24_N_2_O_2_, OMT) is a major active compound of sophora root [22]. It has been reported that OMT has anti-oxidative, anti-inflammation, anti-virus, hepatoprotective, and immunosuppression activities, and currently is extensively employed to treat viral hepatitis, traumatic brain injury, acute pancreatitis, sepsis, and ALI in the clinic [23,24,25]. In the previous study, we have found that OMT, at a low concentration, only inhibits IAV infection in vitro, but cannot in vivo. In the present study, we again determined the effect of OMT on IAV infection at a high concentration in vitro and in vivo, and investigated the mechanism of action of OMT, mainly focusing on the TLRs, PI3K/Akt, MAPK and NF-κB signaling pathways.

## 2. Results

### 2.1. OMT Could Inhibit IAV Replication In Vitro

**Figure 1:**
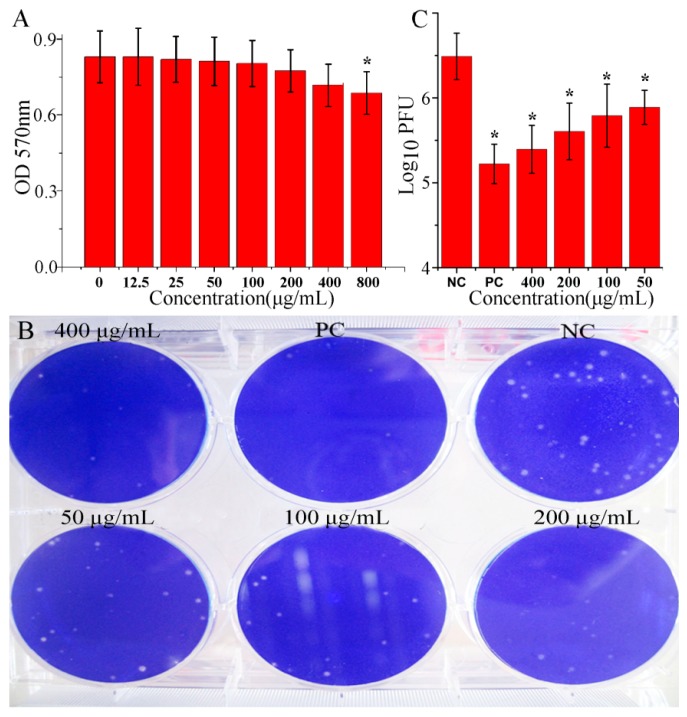
Oxymatrine (OMT) inhibited Influenza A virus (IAV) replication in vitro. (**A**) The cytotoxicity of OMT was determined by a MTT method on A549 cells. Data shown were mean ± SD, *n* = 3, * *p* < 0.05, compared with the 0 μg/mL group; (**B**, **C**) The effect of OMT on IAV replication was determined by a plaque inhibition assay. In the negative control (NC), MDCK cells were infected with IAV (ST169) but not treated with any drugs; in the positive control (PC) and OMT-treated groups, MDCK cells were infected with IAV (ST169) and treated with ribavirin (25 μg/mL) and OMT (400, 200, 100 and 50 μg/mL, respectively), MOI = 0.001, the incubation time was 48 h. Data shown were mean ± SD of five independent experiments. *n* = 5, * *p* < 0.05, compared with the NC group.

Before the experimentation, we had determined the cytotoxicity of OMT on A549 and MDCK cells. The result showed that OMT significantly reduced the viability of A549 cells at the concentration of 800 μg/mL; *y* = −0.061ln(*x*) + 1.0976, *R*^2^ = 0.9923, CC_50_ = 65,986.53 μg/mL (Figure 1A). The cytotoxicity of OMT on MDCK was similar with that on A549 cells (Figure S1). Then through a plaque inhibition assay, we found that OMT could significantly inhibit IAV (ST169, H1N1) replication at the range of 50–400 μg/mL (Figure 1B,C), *y* = 0.273ln(*x*) + 7.0201, *R*^2^ = 0.958, EC_50_ = 17.059 μg/mL, AI (CC_50_/EC_50_) = 3868.1359, and we chose 50 μg/mL as the test concentration for the following pharmacological experiments.

**Figure 2:**
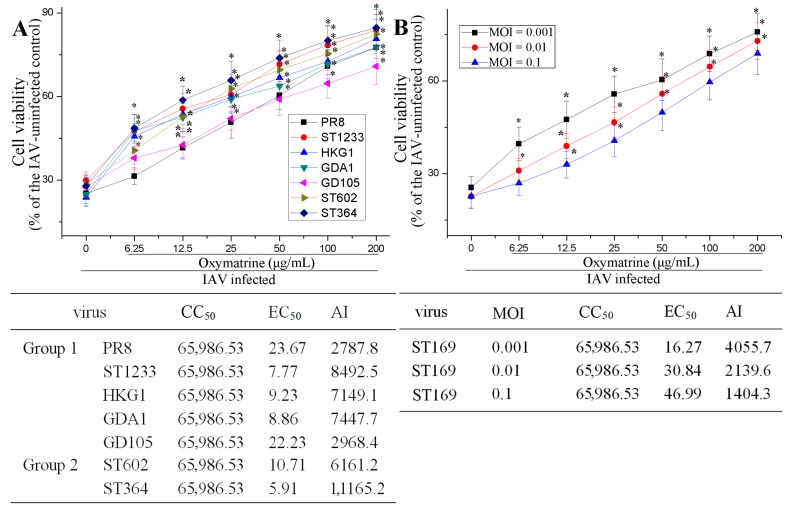
Antiviral activity of OMT on different IAV strains and at different multiplicity of infection (MOI). (**A**) The antiviral activity of OMT on 7 IAV strains, including PR8, ST1233, HKG1, GDA1, GD105, ST602 and ST364, was detected by the SRB method, MOI = 0.001; (**B**) The antiviral activity of OMT against IAV (ST169) infection at different MOI (0.001, 0.01 and 0.1) was detected by the SRB method. The incubation time was 48 h. Data shown were mean ± standard deviation (SD) of five independent experiments. * *p* < 0.05, compared with the 0 μg/mL group.

Besides ST169 (H1N1), OMT also could significantly inhibit PR8 (H1N1), ST1233 (H1N1), HKG1 (H9N2), GDA1 (H9N2), GD105 (H5N1), ST602 (H3N2), and ST364 (H3N2) infection, determined by a sulforhodamine B (SRB) method. The EC_50_ was 23.67, 7.77, 9.32, 8.86, 22.23, 10.71 and 5.91 μg/mL, respectively (Figure 2A). Moreover, OMT also could significantly inhibit ST169 (H1N1) infection at different multiplicity of infection (MOI, 0.001, 0.01 and 0.1) (Figure 2B). Finally, to detect the effect of OMT on virus life cycle, i.e., which steps of IAV replication were inhibited, we further performed a time-of-addition assay, and found that OMT could not directly inactivate IAV and had no significant influence on cells before IAV infection and on IAV adsorption. The inhibition of OMT on IAV replication only occurred during 1–5 h post infection (p.i.) (Figure S2).

### 2.2. OMT Could Inhibit IAV-Induced Activation of TLR3/4/7-Myd88-TRAF6 Pathway

**Figure 3:**
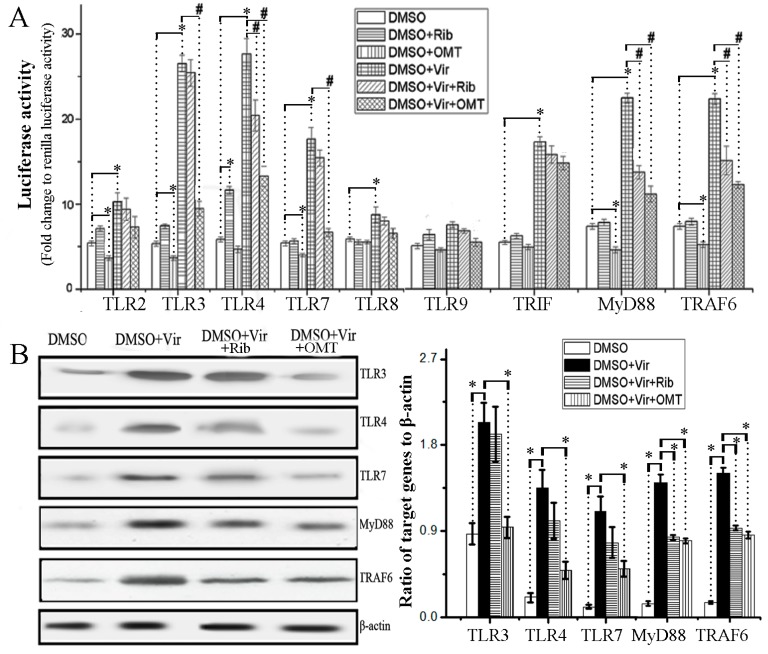
Effects of OMT on the activation of TLRs-MyD88/TRIF-TRAF6 pathway. (**A**) Effects of OMT on the promoter transcription activity of TLRs, TRIF, MyD88, and TRAF6 genes. The luciferase activity was determined following the instruments of Luciferase Reporter Assay Kit. Data shown were mean ± SD of three independent experiments performed in triplicate.* *p* < 0.05, compared with the DMSO group. ^#^ *p* < 0.05, compared with the DMSO + virus (Vir) group; (**B**) Effects of OMT on the expressions of TLR3/4/7, MyD88, and TRAF6 genes were determined by western blotting assay in A549 cells after IAV infection. MOI = 0.001, the incubation time was 48 h. Data shown were mean ± SD of three independent experiments. * *p* < 0.05, compared with the DMSO + virus (Vir) group. Concentrations of ribavirin and OMT were 25 and 50 μg/mL, respectively.

To detect the influence of OMT on the activation of TLR signal pathways, we first constructed the promoter luciferase reporters of human TLR2, TLR3, TLR4, TLR7, TLR8, TLR9, MyD88, TRIF and TRAF6 genes. As shown in Figure 3A, when without IAV infection, OMT alone could significantly decrease the promoter activities of TLR2, TLR3, TLR7, MyD88 and TRAF6 genes; and after IAV infection, OMT also could significantly decrease IAV-induced up-regulations of the promoter activities of TLR3, TLR4, TLR7, MyD88 and TRAF6 genes. Additionally, the result of western blotting assay also showed that OMT could significantly decrease IAV-induced high expressions of TLR3, TLR4, TLR7, MyD88, and TRAF6 genes in A549 cells (Figure 3B).

### 2.3. OMT Could Inhibit IAV-Induced Activation of Akt, ERK1/2, p38 MAPK and NF-κB Pathways

**Figure 4:**
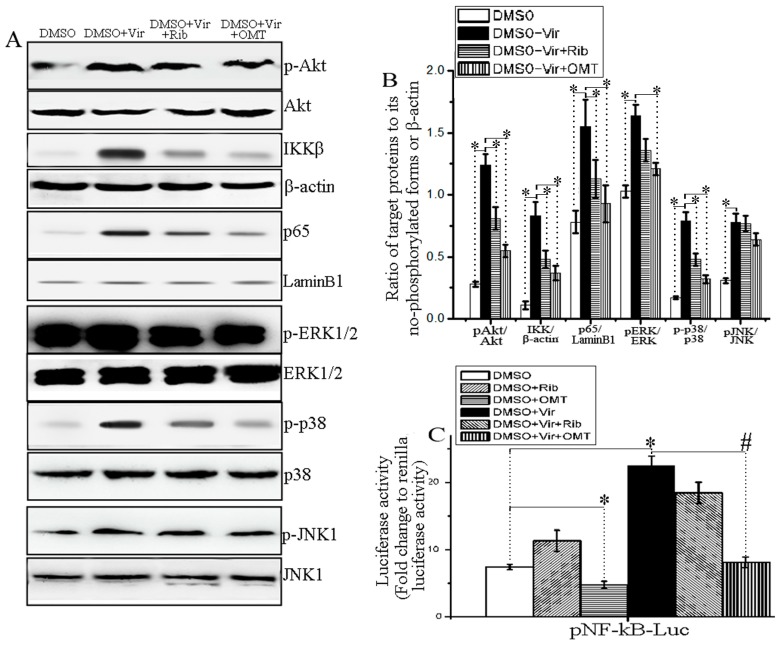
OMT inhibited IAV-induced activation of Akt, ERK1/2, p38 MAPK, and NF-κB signal pathways. (**A**, **B**) Effects of OMT on the phosphorylation of Akt, ERK1/2, p38, JNK, IKKβ and nuclear translocation of NF-κB p65 in A549 cells after IAV infection determined by western blotting assay. Concentrations of ribavirin and OMT were 25 and 50 μg/mL, respectively. MOI = 0.001, the incubation time was 48 h. Data shown were mean ± SD of three independent experiments. * *p* < 0.05 vs. the DMSO + virus group; (**C**) The influence of OMT on the transcriptions of NF-κB downstream target genes was determined by a pNF-κB-luc reporter plasmid assay. The luciferase activity was determined following the instruments of luciferase reporter assay kit. Concentrations of ribavirin and OMT were 25 and 50 μg/mL, respectively. Data shown were mean ± SD of three independent experiments performed in triplicate.* *p* < 0.05, compared with the DMSO group. ^#^ *p* < 0.05, compared with the DMSO + virus (Vir) group.

It has been reported that IAV-induced activations of PI3K/Akt, Raf/MEK/ERK, JNK, p38 MAPK, and NF-κB signaling pathways can support IAV infection [13,18,19,20,21,26,27]. In addition, it has also been reported that OMT can inhibit the activations of TLRs-MyD88-MAPK/NF-κB pathways in brain damage [28], in CCl4-induced cirrhotic rats [29], and in LPS-stimulated BV2 microglial cells [30]; therefore, we also determined the effect of OMT in IAV-infected cells on these signal pathways. As showed in Figure 4A,B, OMT could significantly inhibit IAV-induced phosphorylation of Akt, ERK1/2, p38 MAPK and nuclear translocation of NF-κB p65, but not significantly on IAV-induced phosphorylation of JNK, investigated by a western blotting assay. In addition, to detect the influence of OMT on the transcriptions of NF-κB downstream target genes, we have purchased a pNF-κB-luc reporter plasmid which has 4 duplicated NF-κB response elements (GGGAATTTCC) (D2206, Beyotime Institute of Biotechnology, Shanghai, China). As showed in Figure 4C, OMT could significantly inhibit the transcriptions of pNF-κB-luc reporter plasmid, no matter IAV infection or not.

### 2.4. OMT Might Exert Its Anti-IAV Activity via TLR4, p38 MAPK and NF-κB Signal Pathways

**Figure 5:**
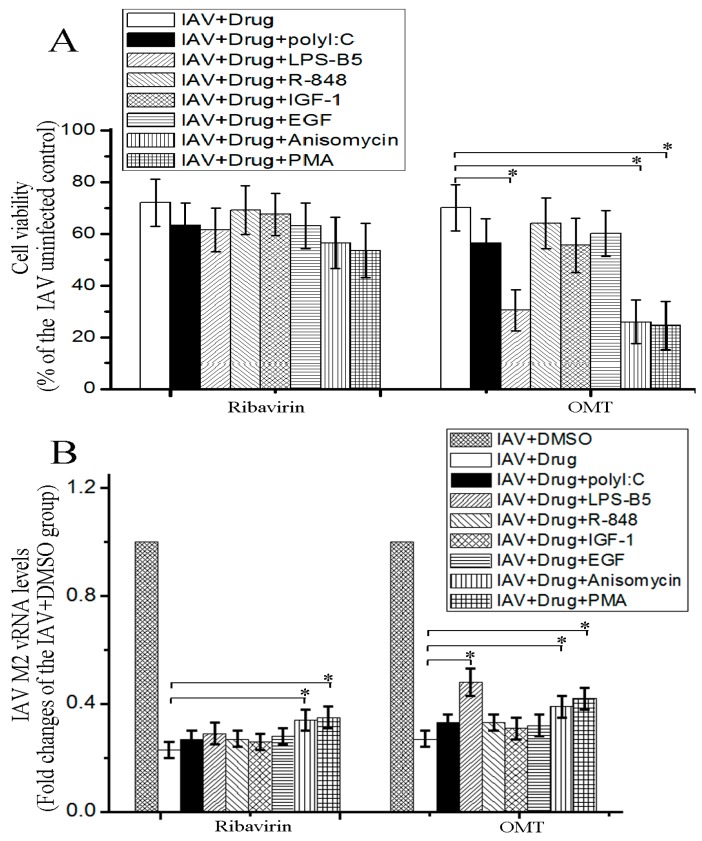
Antagonistic effects of the activators of TLRs, PI-3K/Akt, ERK1/2, p38/JNK, and NF-κB pathways on the anti-IAV activity of OMT. A549 cells were infected with IAV (MOI = 0.001), treated with or without ribavirin (25 μg/mL) and OMT (50 μg/mL), and simultaneously treated with or without TLR3 activator (polyI:C, 100 ng/mL), TLR4 activator LPS-B5 (1 μg/mL), TLR7/8 activator R-848 (10 μg/mL), PI-3K/Akt activator (IGF-1, 100 ng/mL), ERK activator (EGF, 100 ng/mL), p38/JNK activator (Anisomycin, 10 μM), and NF-κB activator (PMA, 1 µg/mL). The test doses of all activators were determined according to previous reports and our preliminary tests. After 48 h, cell viability was determined by the SRB method (**A**) and IAV vRNA levels were determined by a qRT-PCR assay (**B**). Data shown were mean ± SD of three independent experiments performed in triplicate. * *p* < 0.05, compared with the virus + drug control.

To determine the importance of TLRs, PI-3K/Akt, ERK1/2, p38, and NF-κB signal pathways in the anti-IAV activity of OMT, the activators of these signal pathways were used. The result showed that TLR4 activator (LPS-B5), p38 MAPK activator (Anisomycin), and NF-κB activator (PMA) could significantly antagonize the inhibitory effect of OMT on IAV-induced decrease of cell viability determined by the SRB method and significantly counteract the inhibitory effect of OMT on IAV replication determined by a qRT-PCR assay, but the effects of TLR3 activator (polyI:C), TLR7/8 activator (R-848), PI-3K/Akt activator (IGF-1), and ERK1/2 activator (EGF) were not significant (Figure 5).

### 2.5. OMT Could Inhibit IAV-Induced Expressions of Proinflammatory Cytokines and MMP-2/-9

**Figure 6:**
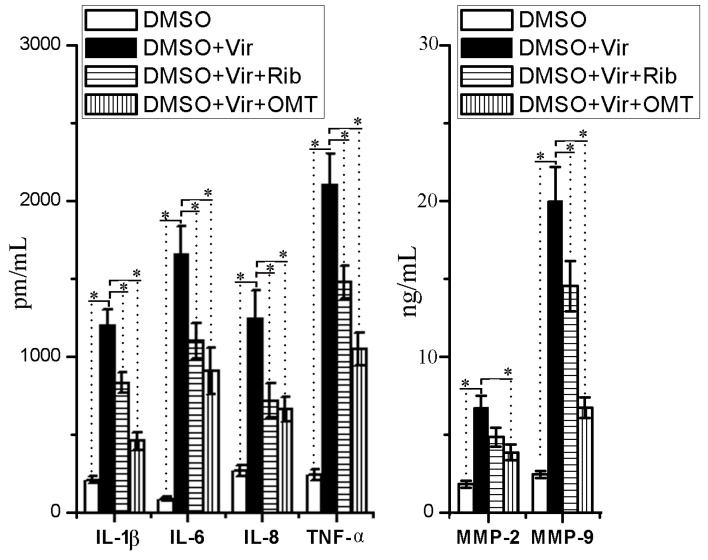
Effects of OMT on the releases of proinflammatory cytokines and MMP-2/-9 after IAV infection. A549 cells were seeded in 6-well plates for 24 h, then infected with or without IAV (ST169, H1N1), MOI = 0.001. In the ribavirin- and OMT-treated groups, the cells were further treated with ribavirin (25 μg/mL) and OMT (50 μg/mL), after 48 h, the supernatants and cells were collected and used in the ELISA assay. Data shown were mean ± SD of three independent experiments with three replicates. * *p* < 0.05, compared with the DMSO + virus (Vir) group.

IAV infection can induce the production of inflammatory cytokines and MMP-2/-9, and high levels of inflammatory cytokines and MMP-2/-9 have been reported to contribute to severe pulmonary histopathological changes after IAV infection [16,17]. In this study, we also determined the influence of OMT on IAV-mediated releases of IL-1β, IL-6, IL-8, TNF-α, MMP-2, and MMP-9 by an ELISA assay. As shown in Figure 6, OMT could significantly inhibit the releases of IL-1β, IL-6, IL-8, TNF-α, MMP-2, and MMP-9 after IAV infection.

### 2.6. OMT Could Ameliorate Lung Inflammation and Improved Pulmonary Histopathological Changes after IAV Infection in Mice

**Figure 7:**
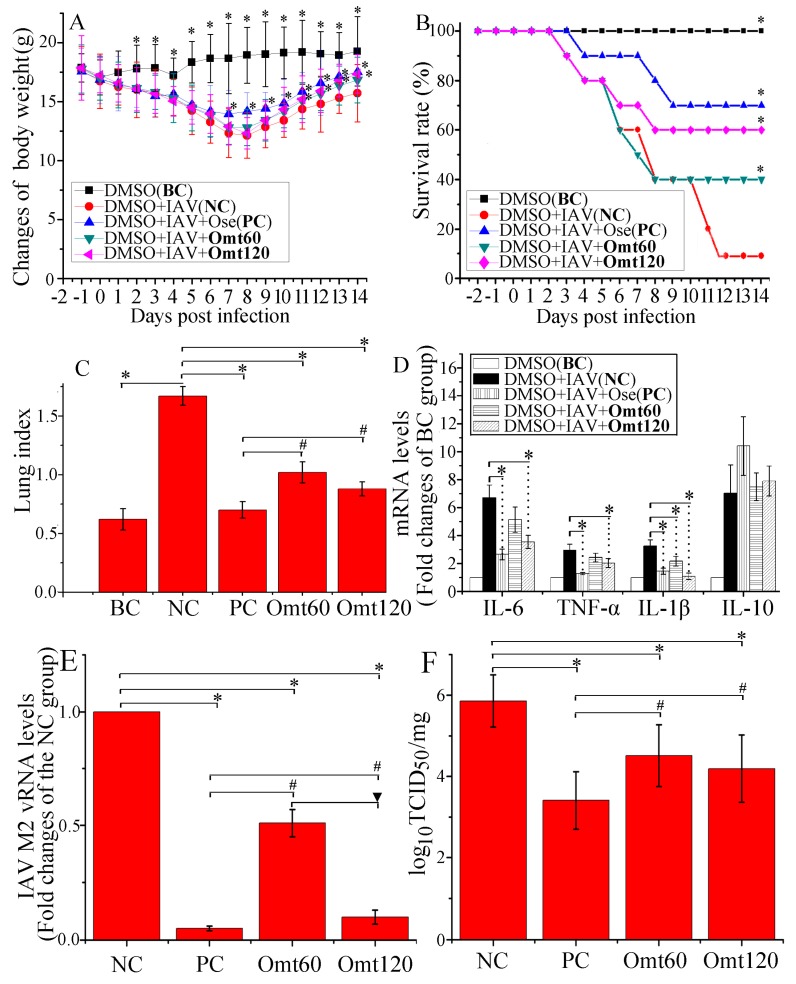
Anti-IAV activity of OMT in vivo. In the blank control (BC), mice were not infected with IAV but shammed intranasally with VGM medium; In the negative control (NC), positive control (PC), and OMT-treated groups (OMT60 and OMT120), mice were intranasally infected with 10× MLD_50_ of IAV (PR8) and treated with 0.5% DMSO, oseltamivir (10 mg/kg/day), and OMT (60 mg/kg/day and 120 mg/kg/day) by oral gavage from day 1 to day 5 p.i., respectively. The changes in body weight (**A**) and survival rates (**B**) were observed for 14 days (*n* = 10). Significant differences in survival rates were analyzed by Kaplan-Meier analysis with Log-rank and Breslow tests; (**C**) The lung index was assessed by determining the percent of lung wet weight (g) to body weight (g) (lung index = lung wet weight (g)/body weight (g) × 100%) at day 6 p.i.; (**D**) The levels of IL-6, TNF-α, IL-1β, and IL-10 in lung homogenates were determined by the qRT-PCR assay at day 6 p.i. (*n* = 6); (**E**, **F**) IAV replication and viral titters in the lungs were determined by the qRT-PCR and TCID_50_ assays at day 6 p.i. (*n* = 6). Data shown were mean ± SD. * *p* < 0.05, compared with the NC (DMSO + virus) group. ^#^ *p* < 0.05, compared with the PC (DMSO + virus + oseltamivir) group. ^▼^ *p* < 0.05, compared with the OMT (60 mg/kg/day) group.

After IAV infection 2 days, the mice started to show disease symptoms of ruffled fur, following labored breathing and loss of body weight. After the 7th and 11th day p.i., oseltamivir (10 mg/kg/day, PC) and OMT (120 mg/kg/day) treatments could significantly increase the body weight of mice infected with IAV(PR8), respectively (Figure 7A). At the 14th day p.i., the survival rates of only-PR8-infected group (NC), oseltamivir (PC), and OMT (120 mg/kg/day, and 60 mg/kg/day)- treated groups were 10%, 70%, 60%, and 40%, respectively. Oseltamivir and OMT could significantly increase the survival rate of mice infected with IAV (PR8) (Figure 7B). In addition, OMT and oseltamivir could significantly reduce the lung index and the transcriptions of IL-6, TNF-α, and IL-1β (Figure 7C,D), and significantly reduce the pulmonary viral load, determined at day 6 p.i. (Figure 7E,F). In addition, comparing with the 60 mg/kg/day- OMT-treated group, OMT at 120 mg/kg/day could further reduce the lung index and viral titers, though not significantly (Figure 7C,F); but in the qRT-PCR assay, IAV M2 vRNA level was significantly decreased in 120 mg/kg/day OMT-treated group (Figure 7E). Furthermore, comparing with oseltamivir (10 mg/kg/day), OMT had significantly less inhibitory effects on the lung index and viral titers.

**Figure 8:**
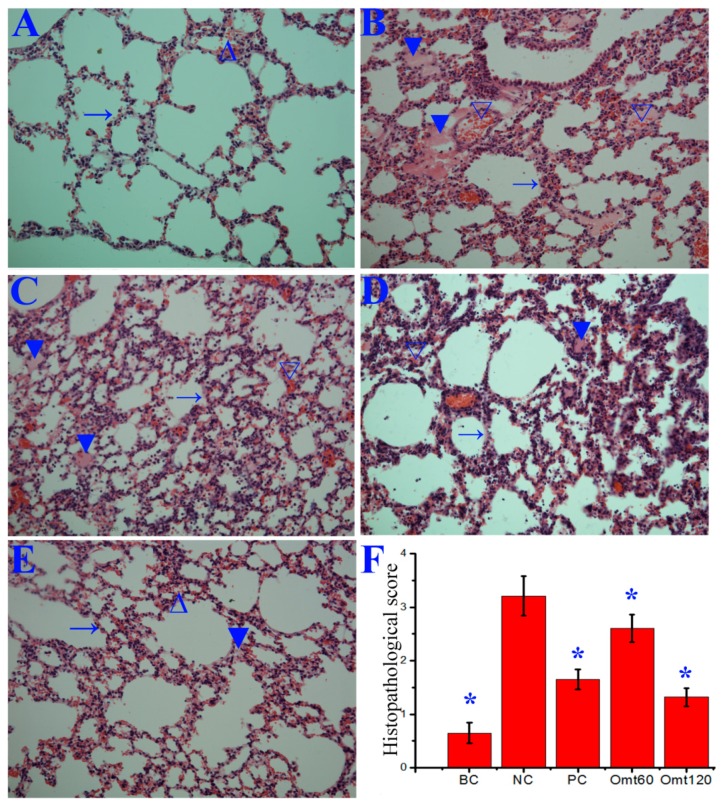
Influence of OMT on pulmonary histopathological changes. Mice were treated as Figure 7 mentioned. At day 6 p.i., six mice from each group were sacrificed. The right lungs were used for haematoxylin and eosin (H&E) staining assay. (**A**) Blank control (BC); (**B**) Negative control (NC); (**C**) Positive control (PC); (**D**, **E**) OMT-treated groups (OMT60 and OMT120, respectively); (**F**) Histopathological score. (→) alveolar wall, (▼) inflammatory exudation, (∆) hemorrhage (erythrocytes). The magnification was 200×. Data shown were mean ± SD. *n* = 6. * *p* < 0.05, compared with the NC control.

Finally, in the histopathological change assay, the lungs of the NC group showed significant alveolar exudation, thickening or destruction of alveolar wall, and alveolar hemorrhage with red blood cells within the alveolar space, while oseltamivir and OMT could significantly inhibit these histopathological changes induced by IAV (PR8) infection, observed at day 6 p.i. (Figure 8).

## 3. Discussion

In fighting IAV infection, traditional Chinese medicine (TCM) has played an important role. In 2009 H1N1 ‘swine flu’ outbreak, Chinese government had released a document entitled “Recommended Schemes for Pandemic Influenza A Diagnoses and Treatments”, which recommended four anti-flu TCM prescriptions [31]. In 2011 and 2018, Chinese National Health and Family Planning Commission had also released a related document for the diagnoses and treatments of ‘flu’, respectively; both of which separately recommended five TCM prescriptions [32,33]. In the past decade, we have always been devoted to the research of anti-IAV drug screening from TCMs. Recently, we have constructed a series of luciferase reporter plasmids based on the gene promoters of the TLRs-MyD88/TRIF-TRAF6 signal pathway, and have screened out several TCMs with excellent anti-IAV activity, and sophora root is one of them. After investigating some previous researches and related literature, we find that sophora root and its major active compound OMT have been used for the treatment of chronic viral hepatitis in the clinic for many years [34]. Moreover, during the pandemic of severe acute respiratory syndrome (SARS) in 2001, Chinese bureau of science and technology has announced that the composite sophora japonica injection (mainly containing OMT) has distinct effects in the treatment of SARS, which has displayed the magical power of OMT to treat systemic inflammatory responses [23]. So we speculate that if OMT can inhibit IAV infection, it may be directly utilized to fight IAV in the clinic. As we initially expect, OMT really can inhibit the replications of IAV, including ST169 (H1N1), PR8 (H1N1), ST1233 (H1N1), HKG1 (H9N2), GDA1 (H9N2), GD105 (H5N1), ST602 (H3N2), and ST364 (H3N2), in vitro and in vivo. Among them, ST169, PR8, ST1233, ST602, and ST364 belong to human IAV, while HKG1, GDA1, and GD105 belong to avian influenza virus.

Then we explore the mechanism of action of OMT on IAV infection. Although TLR signaling pathways play an important role in innate immunity to virus infection, activations of TLR3, TLR4, and TLR7 signaling pathways have been reported to be required for IAV proliferation, and lasting activation of TLR pathways can even support IAV replication and is detrimental to IAV-induced acute pneumonia [11,12,13,15,16]. In the present study, we find that OMT can significantly decrease the expressions of TLR3, TLR4, TLR7, MyD88, and TRAF6 genes after IAV infection, and we preliminarily speculate that OMT may inhibit IAV proliferation and IAV viral pneumonia via inhibiting TLRs signaling pathways.

Activations of PI3K/Akt and the downstream cascades of TLR signaling pathways, such as MAPK and NF-κB, are also reported to be essential for IAV infection. It has been reported that activation of PI3K-AKT-mTOR pathway supports IAV infection [26], and inhibition of Akt will suppress IAV entry and IAV RNP nuclear export [18]. Pleschka S. et al. have showed that activation of Raf/MEK/ERK signaling is a prerequisite for IAV replication, and blockade of ERK pathway can retard RNP export and reduces virus titers [19]. Marjuki H. et al. have showed that IAV-induced activations of ERK and PI3K are required for the fusion of IAV viral particles with the target cells mediated by V-ATPase-dependent intracellular pH change [35]. Guo B. et al. have showed that p38 MAPK is a key determinant of virus entry and tissue tropism [13]. Activation of NF-κB signaling is also a prerequisite for IAV infection, NF-κB inhibitors can specifically diminish IAV vRNA transcription from its cRNA promoter and reduce vRNA synthesis [21]. Our results have shown that OMT can significantly decrease IAV-induced activations of Akt, ERK1/2, p38 MAPK, and NF-κB pathways, which may imply that these signal pathways may also be implicated in the anti-IAV activity of OMT.

To determine the importance of these signal pathways on anti-IAV activity of OMT, the activators of these signal pathways have been used in the present study, and the results show that only TLR4, p38 MAPK, and NF-κB activators can significantly counteract the anti-IAV activity of OMT, including IAV-induced CPE and IAV replication, while TLR3, TLR7/8, PI-3K/Akt and ERK1/2 activators cannot, as showed in Figure 5. So we think that OMT-inhibition on IAV replication may be through inhibiting TLR4, p38 MAPK, and NF-κB signaling pathways. As aforementioned, these pathways have been proved to be essential for IAV replication [13,14,15,21].

IAV infection also induces abnormally elevated expressions of inflammatory cytokines and MMPs, which are reported not only to contribute to severe lung injury, but also to promote IAV proliferation in respiratory tract [16,17,36,37]. OMT is a well-known strong immunosuppressive agent [25], so we have further determined the effect of OMT on the expressions of inflammatory cytokines and MMPs, and find that OMT can significantly suppress IAV-induced releases of IL-1β, IL-6, IL-8, TNF-α, MMP-2, and MMP-9. Moreover, it has been reported that IAV-induced releases of IL-6, IL-8, IL-10, TNF-α, and MMP-9 are MAPK- and NF-κB-dependent [30,36,38], so we speculate that the inhibition of OMT on IAV-induced productions of inflammatory cytokines and MMP-2/-9 may be, at least in part, via inhibiting MAPK and NF-κB pathways.

In fact, it has been reported that OMT can inhibit on the activation of TLRs-MyD88-MAPK/NF-κB pathways and reduce the releases of inflammatory mediators. Fan HG et al. have reported that OMT can protect the brain from damage through down-regulating the TLR4, TLR2, MyD88, and NF-κB pathways [28]. Wen J.B. et al. have indicated that OMT can improve intestinal epithelial barrier function by inhibiting NF-κB-mediated signaling pathway in CCl_4_-induced cirrhotic rats [29]. Dong X.Q. et al. have reported that OMT can inhibit the phosphorylation of ERK, p38, and JNK MAPK, suppress the nuclear translocation of NF-κB p65, decrease the mRNA production of iNOS and COX-2, and finally inhibit the production of NO, PGE2, TNF-α, IL-1β, and IL-6 in LPS-stimulated BV2 microglial cells in a dose-dependent manner [30].

Finally, the in vivo test shows that OMT can inhibit IAV-induced loss of body weight, increase the survival rate, decrease pulmonary edema, the transcriptions of inflammatory cytokines, and pulmonary viral load, and improve histopathological changes. In fact, in the previous study, we have determined the effect of OMT on IAV infection at a lower concentration and find OMT only inhibits IAV infection in vitro, and cannot in vivo. So, we again carry out the present study at a higher concentration in vitro and in vivo. In addition, OMT has also been reported to be able to attenuate oleic acid- induced ALI in mice and cecal ligation and puncture-induced septic shock in rat [23,24], both of which have showed that OMT possesses an excellent activity to inhibit systemic inflammatory responses.

Additionally, anti-inflammatory drugs are divided into steroid and non-steroidal anti-inflammatory drugs. High-dose of steroid anti-inflammatory drugs is usually employed to treat ALI/ARDS, but they often cause serious side effects, such as osteoporosis and vascular necrosis [39]. OMT can exert its anti-inflammatory effect independent of the pituitary-adrenal system, so we speculate that OMT may have more advantageous to treat ALI/ARDS than steroid anti-inflammatory drugs.

## 4. Materials and Methods

### 4.1. Reagents

Oxymatrine (OMT, C_15_H_24_N_2_O_2_, purity > 98%) was purchased from the National Institute for the Control of Pharmaceutical and Biological Products (Beijing, China). MTT (M2003), TPCK-trypsin (4370285), ribavirin (R9644), and sulforhodamine B (SRB, 230162) were purchased from Sigma-Aldrich, Inc. (St. Louis, MO, USA). Minimum essential medium (MEM), lipofectamine 2000 reagent, Pfu DNA polymerase, DNase and TRIzol reagent were purchased from Invitrogen Life Technologies, Inc. (Carlsbad, CA, USA). Luciferase Reporter Assay Kit was bought from BD Biosciences Clontech (Franklin Lakes, NJ, USA). Poly(I:C) (tlrl-pic), LPS-B5 (tlrl-pb5lps), R-848 (tlrl-r848) and PMA (tlrl-pma) were purchased from InvivoGen (San Diego, CA, USA). IGF-1 (8917), EGF (8916), anisomycin (2222), anti-MyD88 (4283), anti-TRAF6 (D-10, sc-8409), anti-p-Akt (10001), anti-Akt (11848), anti-ERK1/2 (8867), anti-p-ERK1/2 (13148), anti-p-JNK (3708), anti-JNK (4671), anti-p-p38 (4092), anti-p38 (14451), anti-p65 (4764), and anti-β-actin (12262) antibodies were bought from Cell Signaling Technology^®^ Inc. Company (Danvers, MA, USA). Anti-TLR3 (sc-517367), anti-TLR4 (sc-293072), anti-TLR7 (H-114, sc-30004), anti-Lamin B1 (sc-56144), secondary horseradish peroxidase-conjugated anti-rabbit or anti-mouse antibodies were purchased from Santa Cruz Biotechnology (Santa Cruz, CA, USA). Pierce™ ECL Plus Western Blotting Substrate (32132) was purchased from Thermo Fisher Scientific™ (Cleveland, OH, USA). All other chemicals and solvents were commercially available and of analytical grade.

### 4.2. Viruses and Cells

The virus stocks of IAV strains A/ShanTou/169/06 (ST169, H1N1), A/PuertoRico/8/34 (PR8, H1N1), A/ShanTou/1233/06 (ST1233, H1N1), A/Quail/HongKong/G1/97 (HKG1, H9N2), A/Chicken/Guangdong/A1/03 (GDA1, H9N2), A/Chicken/Guangdong/1/05 (GD105, H5N1), A/ShanTou/602/06 (ST602, H3N2), and A/ShanTou/364/05 (ST364, H3N2) were prepared in MDCK cells or 10-day-old embryonating eggs. The virus titer was determined by a plaque formation assay [40]. The cytotoxicity of OMT on MDCK and A549 cells was determined by a MTT assay [41]. The concentration of OMT required to lower cell viability by 50% (CC_50_) was calculated by using Origin 8.0 software. All experiments with IAV were performed in a biosafety level 3 (BSL-3) laboratory.

### 4.3. Plasmid Construction

To construct the promoter luciferase reporter plasmids of human TLR2, TLR3, TLR4, TLR7, TLR8, TLR9, MyD88, TRIF, and TRAF6 genes, human TLR2 promoter (NG_016229.1), TLR3 promoter (NT_016354.19), TLR4 promoter (NT_008470.19), TLR7 promoter (NG_012569.1), TLR8 promoter (NG_012882.2), TLR9 promoter (NG_033933.1), MyD88 promoter (NT_022517.18), TRIF promoter (NT_011255.14), and TRAF6 promoter (NT_009237.18) were amplified by Pfu DNA polymerase (invitrogen) using A549 cell genome DNA as template, the PCR productions were cloned into the pGL3-basic vector and named pTlr2-luc, pTlr3-luc, pTlr4-luc, pTlr7-luc, pTlr8-luc, pTlr9-luc, pMyd88-luc, pTrif-luc, and pTraf6-luc, respectively. The primers were presented in Table S1. All constructs were verified by double enzyme digestion (Figure S3) and DNA sequencing assays. The pNF-κB-luc reporter plasmid was purchased from Beyotime Institute of Biotechnology (D2206, Shanghai, China).

### 4.4. Transfection and Luciferase Assay

The A549 cells (1 × 10^6^) were seeded in 6-well plates for 24 h, and then transfected with above mentioned plasmids and renilla luciferase reporter plasmid (internal control) using the lipofectamine 2000 reagent (Invitrogen). After 8 h at 37 °C 5% CO_2_, the cells were washed with phosphate buffered saline (PBS) and infected with IAV (ST169, H1NH) (multiplicity of infection (MOI) = 2.0). After adsorption for 1 h, the cells were washed 3 times with PBS and grown in drug-contained virus growth medium (VGM) (MEM, 0.5μg/mL TPCK-trypsin and 0.125% (*w*/*v*) bovine serum albumin), and incubated for 24 h. The luciferase activity was determined following the instruments of Luciferase Reporter Assay Kit (BD Biosciences Clontech) and the results were presented in fold change after normalization to renilla luciferase activity. DMSO (<0.5%) was used in each group to dissolve the drugs.

### 4.5. Plaque Formation, Plaque Inhibition and Time-of-Addition Assays

Viral titers were determined by a plaque formation assay as previously reported [42]. Plaque inhibition assay was also performed as previously reported [42]. Briefly, A549 cells were infected with IAV (MOI = 0.001) and meanwhile treated with or without different concentrations of OMT for 1 h (adsorption); after washing with PBS 3 times, VGM medium with or without OMT was added again. After 48 h, the cells were lysed by freezing and thawing, after centrifuging at 1000× *g* for 10 min at 4 °C, the supernatants were collected, and the viral titer was determined by a plaque formation assay. The time-of-addition assay contained four tests and performed as previously reported [40]: (a) direct inactivation assay: before infection, IAV virus was incubated with a VGM medium containing OMT (50 μg/mL), after 3 h, IAV virion was gathered by ultra-filtration and washed with PBS 3 times, then used to infect MDCK cells and further cultured for 12 h; (b) influence-on-cell assay: before infection, MDCK cells were incubated with VGM medium containing OMT (50 μg/mL) for 3 h, then the cells were washed with PBS 3 times, infected with normal IAV and further cultured for 12 h; (c) influence-on-viral-adsorption assay: during viral adsorption (1 h), OMT (50 μg/mL) was added, after adsorption, the cells were washed with PBS 3 times and cultured with normal VGM medium for 12 h; and (d) different-time-points post infection (p.i.) assay: after IAV infection, OMT (50 μg/mL) was added at 1, 2, 3, 4, 5, 6, 7 and 8 h p.i., respectively, and further cultured to 12 h p.i. MOI = 2.0. 0.5% DMSO was used as negative control (NC). After 12 h, the cell lysates were gathered and the viral titer was determined by a plaque formation assay.

### 4.6. TCID50 Assay and Antiviral Assay by the Sulforhodamine B (SRB) Method

The stock solution of IAV was first serially diluted with VGM medium. MDCK cells (1 × 10^4^) were seeded in 96-well plates for 24 h and then infected with IAV. After 48 h, the TCID_50_ was calculated following the method of Reed and Muench. Antiviral activities were also evaluated by the SRB method [43,44]. Briefly, MDCK cells were seeded in 96-well plate. 0.09 mL of virus suspension (50 × TCID_50_) and 0.01 mL medium containing the test drug were added. At 48 h, after washing, 100 μL −20 °C 70% acetone was added. After removing acetone, the plates were dried, and added 100 μL 0.4% (*w*/*v*) SRB, after washing, the plates were dried and added 100 μL 10 mM Tris-based solution. OD was read at 562 nm. Three wells were used each for the negative (virus-infected non-drug-treated) and mock controls (non-infected non-drug-treated). 0.5% DMSO was used in each group. Percent protection of OMT, which is positively related to the cell viability, was calculated as the following:

(1) $$\mathrm{Protection}\;\mathrm{of}\;\mathrm{test}\;\mathrm{compound}(\%)=\frac{\bar{OD_{test}}-\bar{OD_{Negative}}}{\bar{OD_{Mock}}-\bar{OD_{Negative}}}\times 100\%$$

Concentration of 50% protection was defined as the EC_50_. Antiviral index (AI) was calculated as CC_50_/EC_50_. The cell viability of negative control was expressed as the percent to the mock group.

### 4.7. Quantitative Real-Time RT-PCR

Quantitative RT-PCR (qRT-PCR) assay was carried out as our previous report [44]. Briefly, total RNA was extracted using Trizol^®^ Plus RNA purification kit (Invitrogen). DNA contamination was removed by adding DNase I (Invitrogen). Total RNA was quantified spectrophotometrically at 260 and 280 nm. First-strand cDNA was synthesized using SuperScript III reverse transcriptase kit (Invitrogen). Real-time PCR reaction mixture contained 2.5 μL cDNA and 200 nM of each primer in SYBR Green I master mix (Invitrogen). The results were expressed as 2^−ΔΔ*C*t^. The primers were presented in Table S2.

### 4.8. Western Blotting Assay

Western blotting was performed as previously reported [43]. Proteins were prepared using RIPA lysis buffer (Shanghai Shenergy Biocolor BioScience and Technology, Shanghai, China). To detect the nuclear translocation of NF-κB p65, nucleic protein was extracted using a EpiQuik Nuclear Extraction Kit (OP-0002-1, Epigentek, Wuhan, China). Protein concentration was determined by BCA assay (ThermoFisher Scientific, Rockford, IL, USA). β-actin was used as an invariant control for total proteins, and lamin B1 was used as a control for nuclear proteins. The results were analyzed with ImageJ v2.1.4.7 software.

### 4.9. ELISA Assay

Cell cultures and lung tissues were collected and frozen at −80 °C. Cytokines and MMPs were quantified by specific ELISA kits. IL-1β (DKW12-3012/-2012), IL-6 (DKW12-1060/-2060), IL-8 (DKW12-1080), and TNF-α (DKW12-1720/-2720) ELISA kits were purchased from Dakewe biological technology co., LTD (Beijing, China). MMP2 (ab100606) and MMP9 (ab100610) ELISA kits were purchased from Abcam Company (Cambridge, UK).

### 4.10. In Vivo Test

All animal experiments were performed in accordance with the ARRIVE guidelines [45]. All experimental procedures were approved by the Institutional Animal Care and Use Committee (IACUC) of Shantou University (Authorization number: SUMC2017-083, 5 March 2017).

Male and female C57BL/6J mice (20 ± 2 g; 6–8 weeks; specified pathogen free (SPF)) were purchased from Shanghai slack laboratory animal co. LTD (Shanghai, China). Animals were housed in a SPF facility containing standard bedding with 12-h light-dark cycles (7 to 19 h, temperature (22 ± 2 °C), humidity (40–70%), controlled ventilation) and fed with standard irradiated pellet food and sterile water ad libitum. Before experiment, mice were fed for 7 days for acclimation. In the preliminary test, twenty mice were used to determine 50% mouse lethal dose (MLD_50_) by the method of Reed and Muench and to determine the test doses of OMT, which were also referred to the previous report [46]. During experiment, 80 mice were randomly divided into 5 groups using the random number table and anesthetized by intraperitoneal injection of ketamine (100 mg/kg). All efforts were made to minimize suffering.

- Blank control (BC, *n* = 16): Mice were not infected with IAV (PR8) but shammed intranasally with VGM medium in a 50 μL volumes, and treated with DMSO (0.5% (*v*/*v*)) by oral gavage.
- Negative control (NC, *n* = 16): Mice were infected intranasally with 10× MLD_50_ of IAV (PR8) in a 50 μL volumes, and treated with DMSO (0.5% (*v*/*v*)) by oral gavage.
- Positive control (PC, *n* = 16): Mice were infected intranasally with 10× MLD_50_ of IAV (PR8) viruses in a 50 μL volumes, and treated with oseltamivir (10 mg/kg/day) by oral gavage.
- OMT-treated groups (OMT60 and OMT120, each group *n* = 16): Mice were infected intranasally with 10× MLD_50_ of IAV (PR8) viruses in a 50 μL volumes, and treated with OMT (60 mg/kg/day, 120 mg/kg/day) by oral gavage, respectively.

DMSO (0.5% (*v*/*v*)), oseltamivir or OMT were given twice a day (at 12-h intervals) for 6 days, starting 24 h after randomly grouping and before virus exposure. The body weights, symptoms, and survivals of ten mice in each group (*n* = 10) were monitored daily for 14 days after virus inoculation. At day 6 p.i., another six mice in each group (*n* = 6) were euthanized by cervical dislocation, the lung index was assessed by determining the percent of lung wet weight (g) to body weight (g) (lung index = lung wet weight (g)/body weight (g) × 100%).Then the collected lungs were separated into two sets, the right lungs were fixed in 10% formalin, and the left lungs were frozen at −80 °C. The left lungs were homogenized in 1 mL of cold MEM medium, and the total protein levels were measured using the BioRad protein assay kit, viral titer and target proteins were determined by TCID_50_, ELISA, or qRT-PCR assays. The unit was corrected for the amount of protein.

To examine pathological changes, the right side of the lung was embedded in paraffin, sectioned at 4 μm for haematoxylin and eosin (H&E) staining. The severity of histological changes was scored according to a semiquantitative scoring system [47], which was further showed in ‘Supplement material of Methods’.

### 4.11. Statistical Analysis

The statistical significance of the comparisons between treated groups was assessed by Student’s *t*-test, one-way ANOVA with post hoc Dunnett’s test, or Kaplan-Meier analysis with Log-rank and Breslow tests using SPSS16.0 software. All values are expressed as mean ± standard deviations (SD). *p* values below 0.05 were considered statistically significant.

## 5. Conclusions

**Figure 9:**
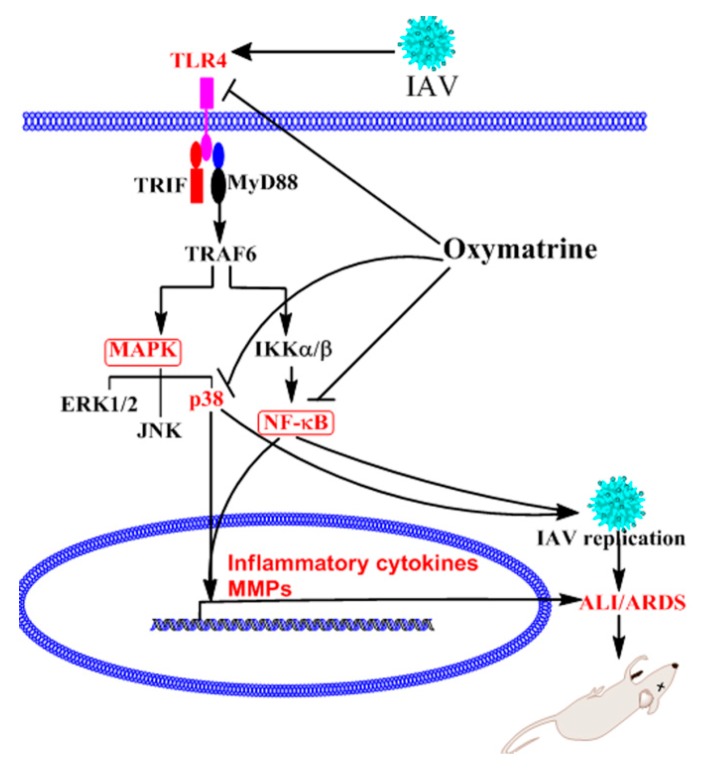
Potential mechanism of OMT to inhibit IAV infection and IAV-induced pneumonia. IAV infection can induce high-expressions of TLR4, MyD88, and TRAF6, phosphorylation of p38 MAPK, and nuclear translocation of NF-κB p65, all of which are essential for IAV proliferation, expressions of inflammatory cytokines and MMPs, and finally lead to ALI/ARDS. The anti-IAV activity of OMT is related to its ability to inhibit IAV-induced activations of TLR4, p38 MAPK, and NF-κB pathways. The “T” bar represents inhibition, while the “arrowhead” represents activation or promotion.

OMT can decrease IAV-induced expressions of TLR4, MyD88, and TRAF6, phosphorylation of p38 MAPK, and nuclear translocation of NF-κB p65. And TLR4, p38 MAPK, and NF-κB activators can antagonize the anti-IAV activity of OMT. So we speculate that the anti-IAV activity of OMT may be related to its ability to inhibit IAV-induced activations of TLR4, p38 MAPK, and NF-κB pathways (Figure 9). Finally, based on the results of our study and other people’s researches [23,24,31], we think that OMT is a promising drug for treating IAV infection and IAV-induced pneumonia.

## Supplementary Materials

Supplementary materials can be found at http://www.mdpi.com/1422-0067/19/4/965/s1.
