# Supplementary material for: Oxymatrine Inhibits Influenza A Virus Replication and Inflammation via TLR4, p38 MAPK and NF-κB Pathways
Source: Int J Mol Sci. 2018 Mar 23;19(4):965. doi: 10.3390/ijms19040965 (PMC5979549; doi:10.3390/ijms19040965)
Supplement: Supplementary file 1 [file ijms-19-00965-s001.zip › Supplement material/Supplement material of Method. Lung injury semiquantitative scoring system.docx]

**Supplement material of Methods**. **Lung injury semiquantitative scoring system**:

0 (no damage),

1 (diffuse reaction in alveolar walls, primarily neutrophilic, no thickening of alveolar walls, congested alveolar space in <1/4 of the field, no hemorrhage);

2 (diffuse presence of neutrophilic and mononuclear in alveolar walls with slight thickening, congested alveolar space in <1/4 – 1/3 of the field, at least five erythrocytes per alveolus in one to five alveoli);

3 (distinct two or three times thickening of alveolar walls due to presence of inflammatory cells, congested alveolar space in 1/3 – 2/3 of the field, at least five erythrocytes in five to ten alveoli);

4 (alveolar wall thickening with up to 50% of lung consolidated, congested alveolar space in >2/3 of the field, at least five erythrocytes in >10 alveoli).

Each slide was assessed by two separate investigators in a blinded manner. To generate the lung injury score, a total of 20 fields were randomly observed at 200× magnification on each slide, and 10 slides were randomly detected for each sample. Representative images are shown. Additionally, to detect infiltrating neutrophilic and mononuclear, 400× magnification was used
