# Supplementary material for: Oxymatrine Inhibits Influenza A Virus Replication and Inflammation via TLR4, p38 MAPK and NF-κB Pathways
Source: Int J Mol Sci. 2018 Mar 23;19(4):965. doi: 10.3390/ijms19040965 (PMC5979549; doi:10.3390/ijms19040965)
Supplement: Supplementary file 1 [file ijms-19-00965-s001.zip › Supplement material/Supplementary Figure S1. The cytotoxicity of OMT on MDCK cells.docx]

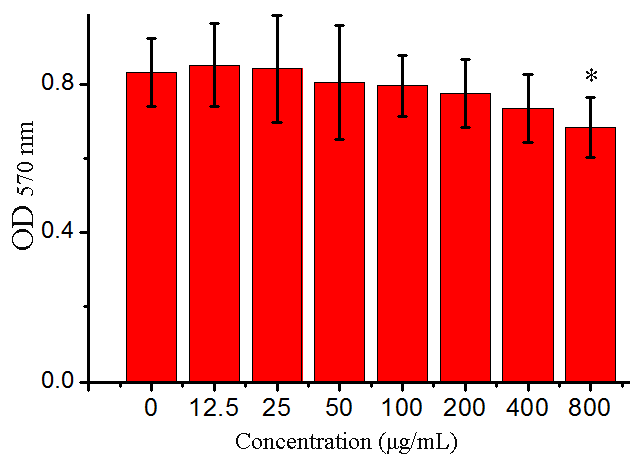


**Supplementary Figure S1.** The cytotoxicity of OMT was determined by a MTT method on MDCK cells. Data shown were mean ± SD, n = 3, * *p* < 0.05, compared with the 0 μg/mL group;
