# Supplementary material for: Oxymatrine Inhibits Influenza A Virus Replication and Inflammation via TLR4, p38 MAPK and NF-κB Pathways
Source: Int J Mol Sci. 2018 Mar 23;19(4):965. doi: 10.3390/ijms19040965 (PMC5979549; doi:10.3390/ijms19040965)
Supplement: Supplementary file 1 [file ijms-19-00965-s001.zip › Supplement material/Supplementary Figure S2. The result of time-of-addition assay.docx]

**
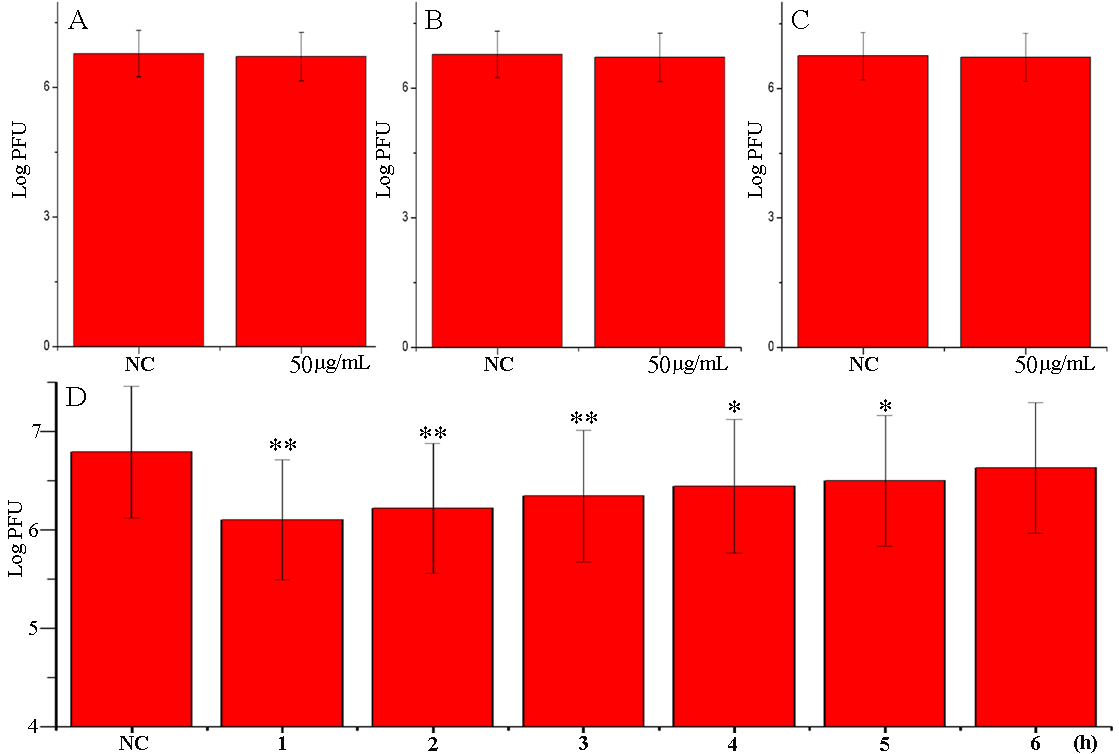
**

**Supplementary Figure S2. The result of time-of-addition assay**. The time-of-addition assay contained four tests: (a) **direct inactivation assay**: before infection, IAV virus was incubated with a VGM medium containing OMT (50 μg/mL), after 3 h, IAV virion was gathered by ultra-filtration and washed with PBS 3 times, then used to infect MDCK cells and further cultured for 12 h; (b) **influence-on-cell assay**: before infection, MDCK cells were incubated with VGM medium containing OMT (50 μg/mL) for 3 h, then the cells were washed with PBS 3 times, infected with normal IAV and further cultured for 12 h; (c) **influence-on-viral-adsorption assay**: during viral adsorption, OMT (50 μg/mL) was added, after adsorption for 1 h, the cells were washed with PBS 3 times and cultured with normal VGM medium for 12 h; and (d) **different-time-points post infection (p.i.) assay**: after IAV infection, OMT (50 μg/mL) was added at 1, 2, 3, 4, 5, 6, 7 and 8 h p.i., respectively, and further cultured to 12 h p.i.. MOI = 2.0. 0.5% DMSO was used as negative control (**NC**). After 12 h, the supernatants were harvested and the viral titer was determined by a plaque formation assay. Data shown were the mean ± SD of five independent experiments.**P* < 0.05 *vs*. the NC group.
