# Supplementary material for: Oxymatrine Inhibits Influenza A Virus Replication and Inflammation via TLR4, p38 MAPK and NF-κB Pathways
Source: Int J Mol Sci. 2018 Mar 23;19(4):965. doi: 10.3390/ijms19040965 (PMC5979549; doi:10.3390/ijms19040965)
Supplement: Supplementary file 1 [file ijms-19-00965-s001.zip › Supplement material/Supplementary Table S1. Primers for cloning human gene promoter.docx]

**Supplementary Table S1. Primers for cloning human gene promoter**

| Promoter | Forward primer 5′-3′ | Reverse primer 5′-3′ | Size (bp) | Genebank NO. |
| --- | --- | --- | --- | --- |
| hTLR2 | AACGGTACCCGGACATACGGACATCTGTGC | AATCTCGAGCTGGGAGAACTCCGAGCAGT | 2828 | NG_016229.1 |
| hTLR3 | GGCGGTACCAGCCATTCATTAGTCAACCAAAG | AGACTCGAGCATTTCATCAGGGAAGTGTGTGGC | 1623 | NT_016354.19 |
| hTLR4 | GGCGGTACCATCCAACATAACTGGTGTCCTT | GTTAAGCTTCACGCAGGAGAGGAAGGCCAT | 694 | NT_008470.19 |
| hTLR7 | GGGCCCTCGAGTTTCTTCTGTTATAAATTCCAGTATTTG | AAATTAAGCTTCCTTTCTTGATGGCATGGAGTGATCC | 967 | NG_012569.1 |
| hTLR8 | CATCTCGAGTGTTTTTGGTTTGCCCATGAAGGCCCAGGC | GCCCAAGCTTCATTCCGTAACTTGCAGCAGCGCAGAATG | 1295 | NG_012882.2 |
| hTLR9 | CCCGGTACCAACAGTTCCCTCTAGGGGCTGAATGTGACC | AATAAGCTTTGCTGGGGGGCAGGGGCTTCTCCAGAGGG | 1018 | NG_033933.1 |
| hMyD88 | TTGCTCGAGCCAGGATAACCAAACATACG | CTTAAGCTTCATGTTGAGAGCAGCCAGG | 2017 | NT_022517.18 |
| hTRIF | AACGGTACCCAGTAGAATTGCTTGGACCC | GTTAAGCTTCGACACCTTCTCCACCTCAG | 625 | NT_011255.14 |
| hTRAF6 | GGAGGTACCGGAGCTTCTAGGGAGGGATA | TTACTCGAGCACTGCTTCCGCCTTCTCT | 1689 | NT_009237.18 |
