# Supplementary material for: Oxymatrine Inhibits Influenza A Virus Replication and Inflammation via TLR4, p38 MAPK and NF-κB Pathways
Source: Int J Mol Sci. 2018 Mar 23;19(4):965. doi: 10.3390/ijms19040965 (PMC5979549; doi:10.3390/ijms19040965)
Supplement: Supplementary file 1 [file ijms-19-00965-s001.zip › Supplement material/Supplementary Table S2. The sequences of primers used in qRT-PCR assay.docx]

**Supplementary Table S2. The sequences of primers used in qRT-PCR assay**

| Genes | Forward primer 5′-3′ | Reverse primer 5′-3′ |
| --- | --- | --- |
| IL-1β | AAGGAGAACCAAGCACGACAAAA | TGGGGAACTCTGCAGACTCAAACT |
| TNF-α | CCAAAGGGATGAGAAGTTCC | CTCCACTTGGTGGTTTGCTA |
| IL-6 | GAGGATACCACTCCCAACAGACC | AAGTGATCATCGTTGTTCATACA |
| IL-10 | CCCAGAAATCAAGGAGCATT | TCACTCTTCACCTGCTCCAC |
| IAV M | AGATGAGTCTTCTAACCGAGGTCG | TGCAAAAACATCTTCAAGTCTCTG |
