# Supplementary figures and images for: Oxymatrine Inhibits Influenza A Virus Replication and Inflammation via TLR4, p38 MAPK and NF-κB Pathways
Source: Int J Mol Sci. 2018 Mar 23;19(4):965. doi: 10.3390/ijms19040965 (PMC5979549; doi:10.3390/ijms19040965)

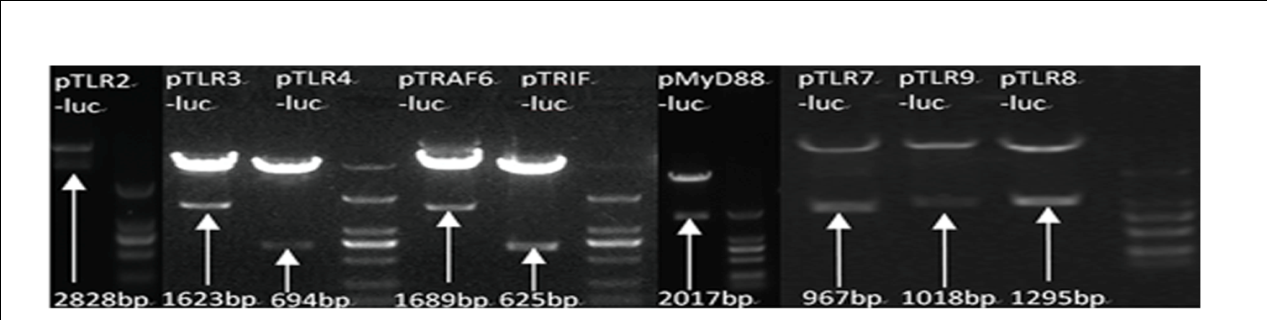


**Supplementary Figure S3**: The results of double enzyme digestion.

Supplement: Supplementary file 1 [file ijms-19-00965-s001.zip › Supplement material/Supplementary Figure S3. The the results of double enzyme digestion.docx]
